# Supplementary material for: Long-term continuous cultivation of Kenyan infant fecal microbiota using the host adapted PolyFermS model
Source: Sci Rep. 2023 Nov 23;13:20563. doi: 10.1038/s41598-023-47131-7 (PMC10667343; doi:10.1038/s41598-023-47131-7)
Supplement: Supplementary file 1 — Supplementary Information. [file 41598_2023_47131_MOESM1_ESM.docx]

**Long-term continuous cultivation of Kenyan infant fecal microbiota using the host adapted PolyFermS model**

Carole Rachmühl^1^, Christophe Lacroix^1*^, Paula Momo Cabrera^1^, Annelies Geirnaert^1*^

1 Laboratory of Food Biotechnology, Institute of Food, Nutrition and Health, ETH Zurich, Zurich, Switzerland

*Correspondence: [annelies.geirnaert@hest.ethz.ch](mailto:annelies.geirnaert@hest.ethz.ch) and [christophe.lacroix@hest.ethz.ch](mailto:christophe.lacroix@hest.ethz.ch)

**Supplementary table 1.** Quantification of key bacterial taxa in fecal inoculum and corresponding *in vitro* Kenyan infant fecal microbiota continuous cultivated with different doses of FOS.

| **Infant** | **Sample** | **Total bacteria** | **Firmicutes** | ***Lachno-***  ***spiraceae*** | ***Rumino-***  ***coccaceae*** | ***Veillonella*** | **LLP** | ***Bifido-bacterium*** | ***Entero-***  ***bacteriaceae*** | ***Bacteroides*** |
| --- | --- | --- | --- | --- | --- | --- | --- | --- | --- | --- |
| **01** | Feces | 10.30 | 9.66 | 7.60 | 6.83 | 7.68 | 6.71 | 9.68 | 8.05 | 8.23 |
|  | Reactor FOS **4 g/L** | 10.50±0.09 | 9.62±0.09 | 8.67±0.12 | 8.37±0.06 | 7.25±0.14 | 7.37±0.08 | 9.18±0.06 | 7.64±0.07 | 9.81±0.08 |
|  | Reactor FOS **8 g/L** | 10.47±0.05 | 9.73±0.04 | 7.80±0.21 ** | 9.44±0.06 **** | 7.14±0.09 | 6.51±0.08 *** | 8.19±0.25 ** | 7.33±0.07 ** | 9.35±0.08 ** |
| **02** | Feces | 10.40 | 9.43 | 5.92 | 5.46 | 8.87 | 6.68 | 9.94 | 8.06 | 5.57 |
|  | Reactor FOS **4 g/L** | 10.49±0.05 | 9.59±0.10 | 8.85±0.06 | 7.68±0.08 | 8.03±0.37 | 7.14±0.07 | 9.33±0.07 | 6.45±0.38 | 9.70±0.04 |
|  | Reactor FOS **8 g/L** | 10.55±0.03 | 9.79±0.03 * | 8.09±0.13 *** | 9.50±0.06 **** | 8.35±0.29 | 7.15±0.14 | 8.73±0.06 *** | 6.96±0.12 | 9.44±0.03 |
| **03** | Feces | 10.97 | 9.93 | 9.02 | 8.89 | 10.31 | 8.35 | 10.05 | 8.90 | 9.97 |
|  | Reactor **w/o** FOS | 10.04±0.10 a** | 9.47±0.07 | 8.60±0.18 a** | 8.24±0.11 a** | 6.94±0.19 a**** | 6.42±0.23 a | 7.66±0.18 | 7.04±0.31 | 9.33±0.05 |
|  | Reactor FOS **1 g/L** | 10.06±0.06 a* | 9.44±0.06 | 8.63±0.14 a** | 8.17±0.08 a*** | 7.23±0.16 a*** | 6.90±0.28 b** | 7.68±0.12 | 6.75±0.16 | 9.40±0.04 |
|  | Reactor FOS **4 g/L** | 10.31±0.02 b | 9.57±0.04 | 9.10±0.09 b | 8.73±0.09 b | 7.85±0.30  b | 6.82±0.28 b*** | 7.95±0.24 | 6.94±0.26 | 9.57±0.04 |

The numbers represent log_10_ 16S rRNA gene copies / g feces or mL reactor effluent for total bacteria or log_10_ bacteria / g feces or mL reactor effluent for the specific targets. Mean ± SD of the last three days of fermentation is shown for *in vitro* microbiota (reactor). Mean of technical triplicates is shown for feces. BDL: below detection limit, LLP: *Lactobacillus*/*Leuconostoc*/*Pediococcus*, w/o: without. Significant differences between FOS treatments are indicated. For infant 03, different letters indicate significant differences. *p<0.05, **p<0.01, ***p<0.001, ****p<0.0001


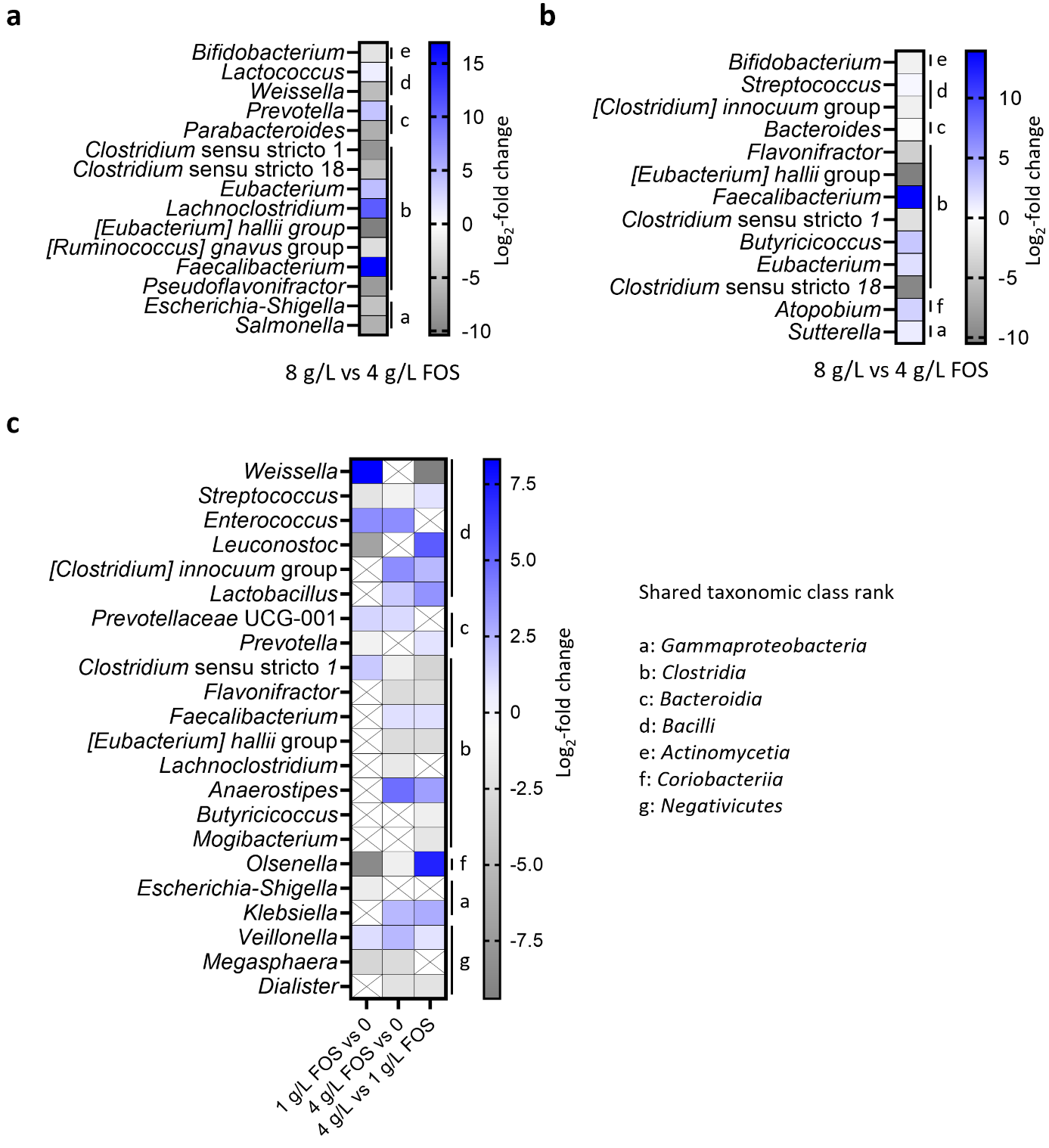
**Supplementary figure 1.** Differential abundance analysis at genus level of *in vitro* microbiota (IVM) exposed to different doses of FOS (1, 4 and 8 g/L). Heatmap showing significant (p<0.05) log_2_-fold changes in abundance for IVM 01 (**a**), IVM 02 (**b**) and IVM 03 (**c**). The genera are grouped based on shared taxonomic class rank. A cross indicates no significant changes in relative abundance of the respective genus.


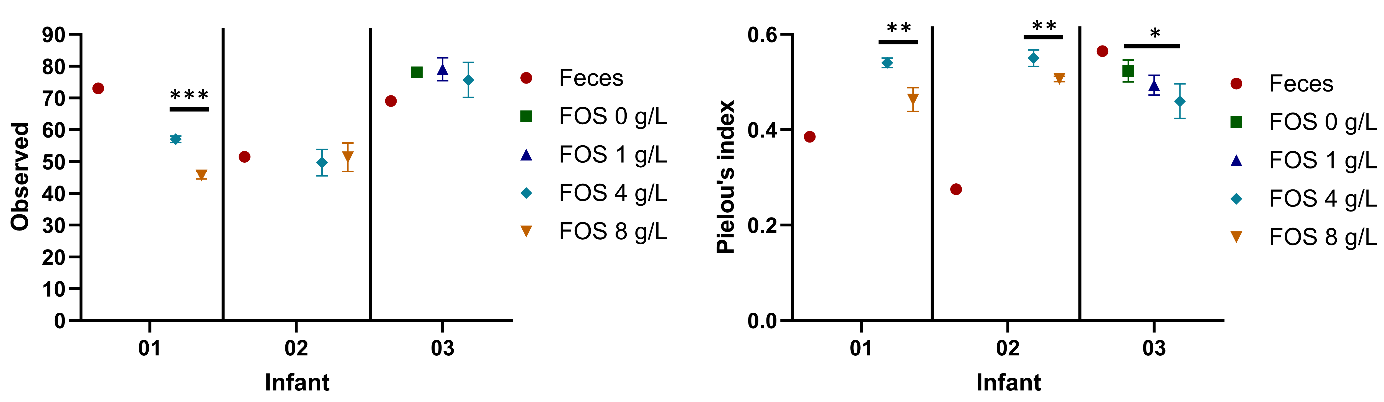
**Supplementary figure 2.** Alpha diversity of Kenyan infant feces and *in vitro* microbiota exposed to different doses of FOS (1, 4 and 8 g/L). Richness is indicated by number of observed ASVs and evenness by Pielou’s index. Mean ± SD of three consecutive fermentation days is shown. *p<0.05, **p<0.01, ***p<0.001

**Supplementary table 2.** Quantification of key bacterial taxa in fecal inoculum and corresponding *in vitro* Kenyan infant fecal microbiota continuously cultivated at different pH.

| **Infant** | **Sample** | **Total bacteria** | **Firmicutes** | ***Lachno-***  ***spiraceae*** | ***Rumino-coccaceae*** | ***Veillonella*** | **LLP** | ***Bifido-***  ***bacterium*** | ***Entero-bacteriaceae*** | ***Bacteroides*** | |
| --- | --- | --- | --- | --- | --- | --- | --- | --- | --- | --- | --- |
| **04** | Feces | 10.57 | 9.95 | 7.59 | 6.51 | 8.49 | 9.48 | 10.02 | 9.42 | 8.87 | |
|  | Reactor pH **5.8** | 10.25±0.08 | 9.59±0.02 | 8.30±0.07 | 5.13±0.08 | BDL | 9.69±0.05 | 9.25±0.06 | 6.77±0.05 | 9.04±0.05 | |
|  | Reactor pH **6.3** | 10.41±0.06 | 9.79±0.05 ** | 9.10±0.12 *** | 5.16±0.11 | BDL | 7.49±0.04 **** | 9.13±0.05 * | 8.39±0.13 **** | | 9.56±0.04 *** |
| **05** | Feces | 10.08 | 9.46 | 7.38 | BDL | 8.64 | 7.51 | 9.68 | 7.97 | 7.40 | |
|  | Reactor pH **5.8** | 10.35±0.08 | 9.52±0.13 | 9.06±0.24 | BDL | 8.44±0.40 | 7.72±0.42 | 7.90±0.27 | 5.81±0.34 | 9.76±0.05 | |
|  | Reactor pH **6.3** | 10.43±0.10 | 9.69±0.07 | 9.60±0.03 * | 8.82±0.24 | 7.34±0.49 | 5.86±0.28 ** | 7.31±0.17 * | 5.95±0.44 | 9.65±0.07 | |
| **06** | Feces | 10.84 | 9.86 | 7.85 | BDL | 9.60 | 8.24 | 10.35 | 8.63 | 6.35 | |
|  | Reactor pH **5.8** | 10.45±0.03 | 9.44±0.08 | BDL | BDL | 9.54±0.13 | 7.66±0.20 | 9.71±0.05 | 7.42±0.26 | 5.50±0.65 | |
|  | Reactor pH **6.3** | 10.36±0.07 | 9.29±0.19 | BDL | BDL | 7.73±0.17 **** | 7.12±0.18 * | 8.48±0.04 **** | 6.94±0.15 * | 8.10±0.10 * | |
| **07** | Feces | 10.10 | 9.25 | 5.51 | 5.51 | 7.40 | 7.01 | 9.19 | 8.55 | 7.87 | |
|  | Reactor pH **5.8** | 10.61±0.04 | 9.43±0.09 | 8.89±0.05 | 7.78±0.05 | 7.86±0.14 | 7.31±0.14 | 8.36±0.08 | 7.05±0.46 | 9.98±0.03 | |
|  | Reactor pH **6.3** | 10.59±0.10 | 9.43±0.08 | 8.80±0.11 | 7.60±0.05 ** | 7.46±0.27 | 6.53±0.16 ** | 8.14±0.19 | 6.28±0.13 * | 9.97±0.08 | |

The numbers represent log_10_ 16S rRNA gene copies / g feces or mL reactor effluent for total bacteria or log_10_ bacteria / g feces or mL reactor effluent for the other targets. Mean ± SD of the last three days of fermentation is shown for *in vitro* microbiota (reactor). Mean of technical triplicates is shown for feces. BDL: below detection limit, LLP: *Lactobacillus*/*Leuconostoc*/*Pediococcus*. Significant differences between different cultivation pH are indicated. *p<0.05, **p<0.01, ***p<0.001, ****p<0.0001


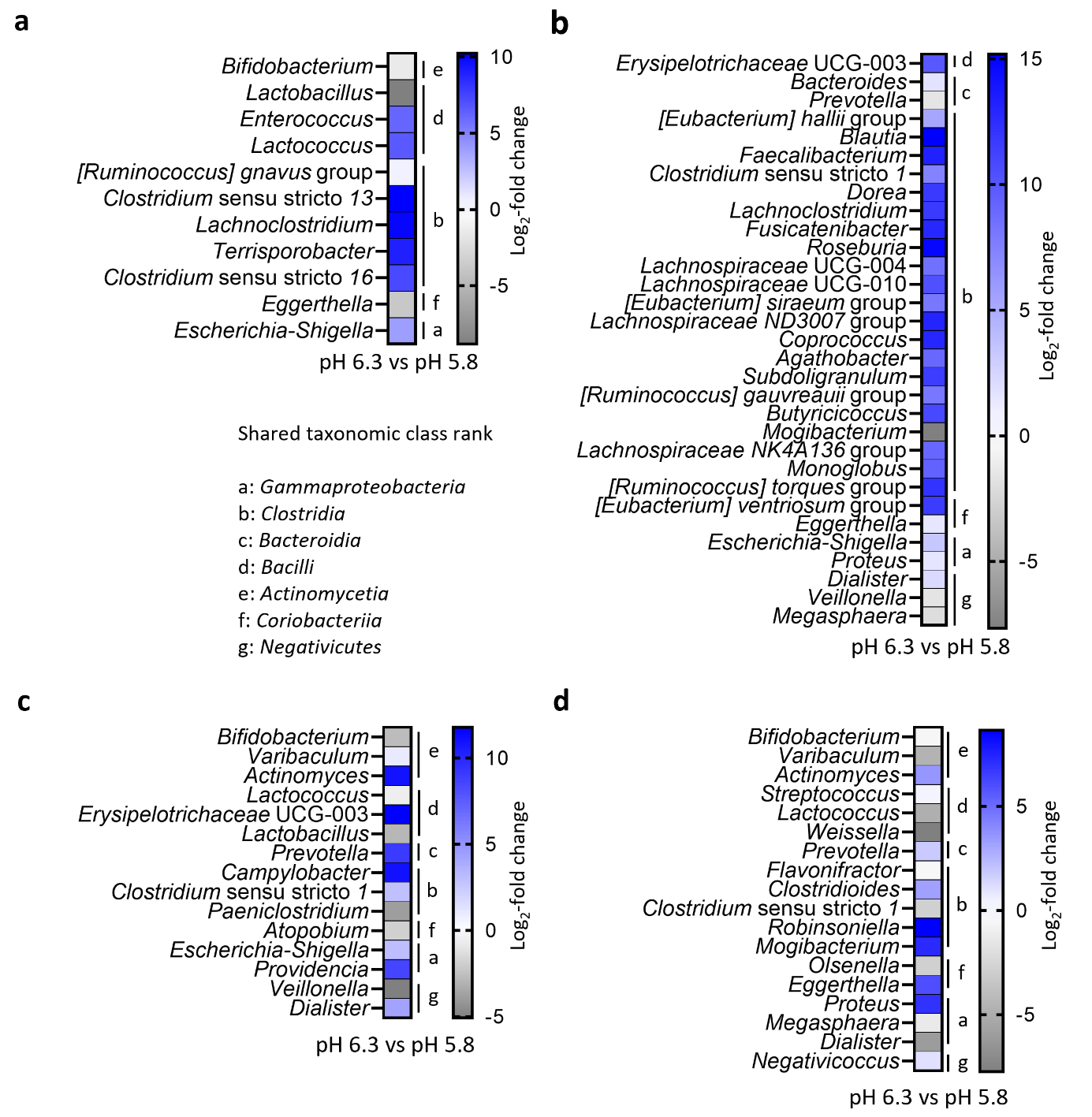
**Supplementary figure 3.** Differential abundance analysis at genus level of *in vitro* microbiota (IVM) exposed to different cultivation pH (5.8 and 6.3). Heatmap showing significant (p<0.05) log_2_-fold changes in abundance for IVM 04 (**A**), IVM 05 (**B**), IVM 06 (**C**) and IVM 07 (**D**). The genera are grouped based on shared taxonomic class rank.

**
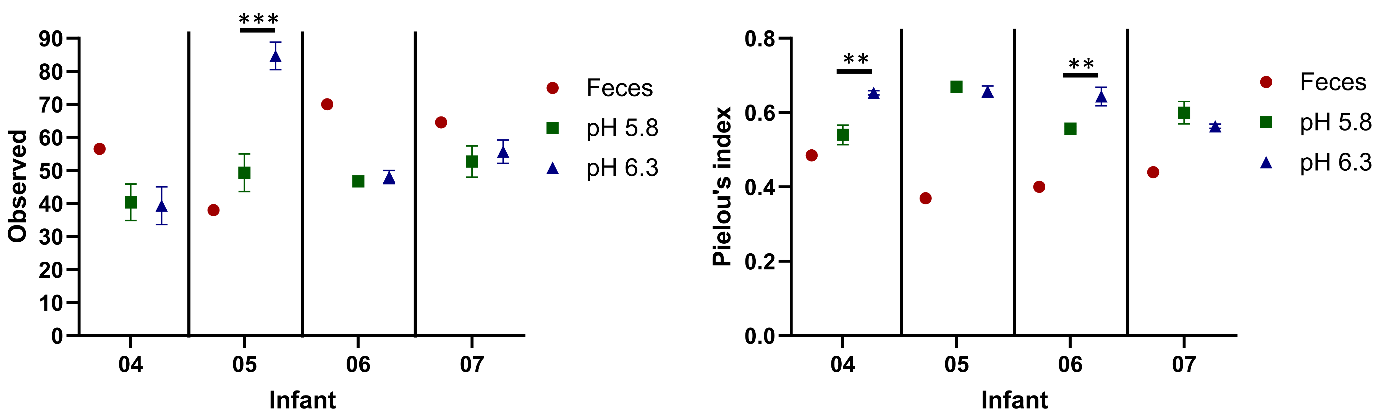
Supplementary figure 4.** Alpha diversity of Kenyan infant feces and *in vitro* microbiota cultivated at different pH (5.8 and 6.3). Richness is indicated by number of observed ASVs and evenness by Pielou’s index. Mean ± SD of three consecutive fermentation days is shown. *p<0.05, **p<0.01, ***p<0.001


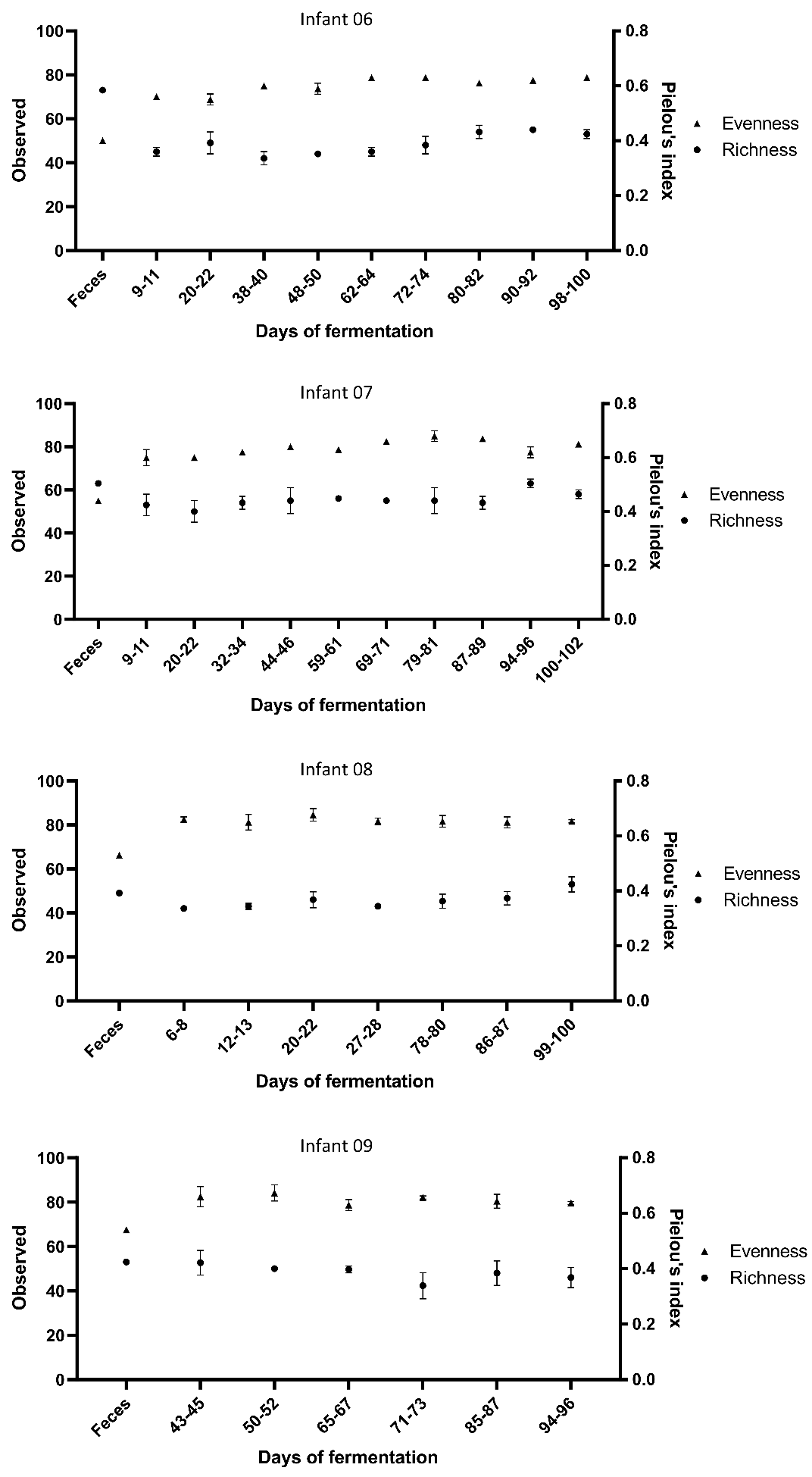
**Supplementary figure 5.** Alpha diversity of four Kenyan infant gut microbiota over long-term continuous fermentation. Number of observed ASVs (richness) and Pielou’s index (evenness) are shown for feces and over the course of fermentation at pH 5.8 and FOS 1 g/L. Mean ± SD of three consecutive days of fermentation is shown.

**Supplementary table 3.** Primers used for quantitative PCR. All standards were constructs of pGEM-T Easy vector containing the 16S rRNA gene of the specific target.

| Target | Primer | Sequence (5’-3’) | Standard  16SrRNA gene | Amplicon length | Ref. | Copy no. |
| --- | --- | --- | --- | --- | --- | --- |
| Total bacteria | Eub338F  Eub518R | ACT CCT ACG GGA GGC AGC AG ATT ACC GCG GCT GCT GG | *E. coli* | 200 bp | [1] | - |
| *Veillonella* | Vspp - F  Vspp - R | AYC AAC CTG CCC TTC AGA CGT CCC GAT TAA CAG AGC TT | *V. rattii* | 343 bp | [2] | 4 |
| *Bacteroides* | Bac303F  Bfr-Femrev | GAA GGT CCC CCA CAT TG CGC KAC TTG GCT GGT TCA G | *B. thetaio-taomicron* | 103 bp | [3] | 3 |
| *Lactobacillus*/*Pediococcus* /*Leuconostoc* | F_Lacto 05  R_Lacto 04 | AGC AGT AGG GAA TCT TCC A CGC CAC TGG TGT TCY TCC ATA TA | *L. delbrueckii* | 375 bp | [4] | 5 |
| *Entero*-*bacteriaceae* | Eco1457F  Eco1652R | CAT TGA CGT TAC CCG CAG AAG AAG C CTC TAC GAG ACT CAA GCT TGC | *E. coli* | 195 bp | [5] | 7 |
| *Bifido*-*bacterium* | Bif F  Bif R | TCG CGT CYG GTG TGA AAG CCA CAT CCA GCR TCC AC | *B. adolescentis* | 243 bp | [2] | 3 |
| Firmicutes | Firm934F  Firm1060R | GGA GYA TGT GGT TTA ATT CGA AGC A AGC TGA CGA CAA CCA TGC AC | *R. intestinalis* | 126 bp | [6] | 7 |
| *Lachno*-*spiraceae* (Clostridial Cluster XIVa) | Ccoc-F  Ccoc-R | AAA TGA CGG TAC CTG ACT AA CTT TGA GTT TCA TTC TTG CGA A | *R. intestinalis* | 438-441 bp | [7] | 5 |
| *Rumino*-  *coccaceae* (Clostridial cluster IV) | Clep866mF  Clep1240mR | TTA ACA CAA TAA GTW ATC CAC CTG G ACC TTC CTC CGT TTT GTC ACC | *F. prausnitzi* | 314 bp | [3] | 4 |

**References**

1. Fierer, N., Jackson, J. A., Vilgalys, R. & Jackson, R. B. Assessment of soil microbial community structure by use of taxon-specific quantitative PCR assays. *Appl. Environ. Microbiol.* **71**, 4117–4120 (2005).

2. Rinttilä, T., Kassinen, A., Malinen, E., Krogius, L. & Palva, A. Development of an extensive set of 16S rDNA-targeted primers for quantification of pathogenic and indigenous bacteria in faecal samples by real-time PCR. *J. Appl. Microbiol.* **97**, 1166–1177 (2004).

3. Ramirez-Farias, C. *et al.* Effect of inulin on the human gut microbiota: stimulation of Bifidobacterium adolescentis and Faecalibacterium prausnitzii. *Br. J. Nutr.* **101**, 541–550 (2008).

4. Furet, J.-P. *et al.* Comparative assessment of human and farm animal faecal microbiota using real-time quantitative PCR. *FEMS Microbiol. Ecol.* **68**, 351–362 (2009).

5. Bartosch, S., Fite, A., Macfarlane, G. T. & McMurdo, M. E. T. Characterization of bacterial communities in feces from healthy elderly volunteers and hospitalized elderly patients by using real-time PCR and effects of antibiotic treatment on the fecal microbiota. *Appl. Environ. Microbiol.* **70**, 3575–3581 (2004).

6. Guo, X. *et al.* Development of a real-time PCR method for Firmicutes and Bacteroidetes in faeces and its application to quantify intestinal population of obese and lean pigs. *Lett. Appl. Microbiol.* **47**, 367–373 (2008).

7. Sagheddu, V., Patrone, V., Miragoli, F., Puglisi, E. & Morelli, L. Infant Early Gut Colonization by Lachnospiraceae: High Frequency of Ruminococcus gnavus. *Front. Pediatr.* **4**, 1 (2016).
